# Performance of Three Anti-SARS-CoV-2 Anti-S and One Anti-N Immunoassays for the Monitoring of Immune Status and Vaccine Response

**DOI:** 10.3390/v16020292

**Published:** 2024-02-14

**Authors:** Y. Victoria Zhang, Attila Kumanovics, Joesph Wiencek, Stacy E. F. Melanson, Tanzy Love, Alan H. B. Wu, Zhen Zhao, Qing H. Meng, David D. Koch, Fred S. Apple, Caitlin R. Ondracek, Robert H. Christenson

**Affiliations:** 1 Department of Pathology and Laboratory Medicine, University of Rochester Medical Center, Rochester, NY 14642, USA; 2 Department of Laboratory Medicine and Pathology, Mayo Clinic, Rochester, MN 55905, USA; kumanovics.attila@mayo.edu; 3 Department of Pathology, Microbiology and Immunology, Vanderbilt School of Medicine, Nashville, TN 37240, USA; joe.wiencek@vumc.org; 4 Department of Pathology, Brigham and Women’s Hospital, Boston, MA 02115, USA; semelanson@bwh.harvard.edu; 5 Harvard Medical School, Boston, MA 02115, USA; 6 Department of Biostatistics and Computational Biology, University of Rochester, Rochester, NY 14642, USA; tanzy_love@urmc.rochester.edu; 7 Department of Laboratory Medicine, University of California, San Francisco, CA 94143, USA; alan.wu@ucsf.edu; 8 Department of Laboratory Medicine and Pathology, Weill Cornell Medicine, New York, NY 10065, USA; zhz9010@med.cornell.edu; 9 Department of Laboratory Medicine, The University of Texas MD Anderson Cancer Center, Houston, TX 77030, USA; qhmeng@mdanderson.org; 10 Department of Pathology and Laboratory Medicine, Emory University, Atlanta, GA 30303, USA; ddkoch@emory.edu; 11 Department of Laboratory Medicine and Pathology, Hennepin Healthcare/Hennepin County Medical Center, Minneapolis, MN 55404, USA; apple004@umn.edu; 12 Hennepin Healthcare Research Institute, Minneapolis, MN 55404, USA; 13 Association for Diagnostics & Laboratory Medicine, Washington, DC 22203, USA; condracek@myadlm.org; 14 Department of Pathology, University of Maryland School of Medicine, Baltimore, MD 21201, USA

**Keywords:** COVID-19, SARS-CoV-2, anti-S antibody, anti-N antibody, vaccine

## Abstract

This study aimed to evaluate and compare the performance of three anti-S and one anti-N assays that were available to the project in detecting antibody levels after three commonly used SARS-CoV-2 vaccines (Pfizer, Moderna, and Johnson & Johnson). It also aimed to assess the association of age, sex, race, ethnicity, vaccine timing, and vaccine side effects on antibody levels in a cohort of 827 individuals. In September 2021, 698 vaccinated individuals donated blood samples as part of the Association for Diagnostics & Laboratory Medicine (ADLM) COVID-19 Immunity Study. These individuals also participated in a comprehensive survey covering demographic information, vaccination status, and associated side effects. Additionally, 305 age- and gender-matched samples were obtained from the ADLM 2015 sample bank as pre-COVID-19-negative samples. All these samples underwent antibody level analysis using three anti-S assays, namely Beckman Access SARS-CoV-2 IgG (Beckman assay), Ortho Clinical Diagnostics VITROS Anti-SARS-CoV-2 IgG (Ortho assay), Siemens ADVIA Centaur SARS-CoV-2 IgG (Siemens assay), and one anti-N antibody assay: Bio-Rad Platelia SARS-CoV-2 Total Ab assay (BioRad assay). A total of 827 samples (580 COVID-19 samples and 247 pre-COVID-19 samples) received results for all four assays and underwent further analysis. Beckman, Ortho, and Siemens anti-S assays showed an overall sensitivity of 99.5%, 97.6%, and 96.9%, and specificity of 90%, 100%, and 99.6%, respectively. All three assays indicated 100% sensitivity for individuals who received the Moderna vaccine and boosters, and over 99% sensitivity for the Pfizer vaccine. Sensitivities varied from 70.4% (Siemens), 81.5% (Ortho), and 96.3% (Beckman) for individuals who received the Johnson & Johnson vaccine. BioRad anti-N assays demonstrated 46.2% sensitivity and 99.25% specificity based on results from individuals with self-reported infection. The highest median anti-S antibody levels were measured in individuals who received the Moderna vaccine, followed by Pfizer and then Johnson & Johnson vaccines. Higher anti-S antibody levels were significantly associated with younger age and closer proximity to the last vaccine dose but were not associated with gender, race, or ethnicity. Participants with higher anti-S levels experienced significantly more side effects as well as more severe side effects (e.g., muscle pain, chills, fever, and moderate limitations) (*p* < 0.05). Anti-N antibody levels only indicated a significant correlation with headache. This study indicated performance variations among different anti-S assays, both among themselves and when analyzing individuals with different SARS-CoV-2 vaccines. Caution should be exercised when conducting large-scale studies to ensure that the same platform and/or assays are used for the most effective interpretation of the data.

## 1. Introduction

The emergence of the severe acute respiratory syndrome coronavirus 2 (SARS-CoV-2) in late 2019 led to a global pandemic that has posed significant challenges to public health worldwide [1,2]. Ongoing research is dedicated to understanding the nature of the disease, its multifaceted impacts on daily life, public health considerations, and preventive measures. Serological assays that were designed to measure SARS-CoV-2 antibodies have garnered attention from both the scientific community and the public throughout the pandemic. Many serological assays have been developed and gained the FDA Emergency Use Authorization (EUA) [3,4,5]. However, due to the expedited approval process, the performances and utilities of these assays were not thoroughly established. 

Understanding the comparative performance of these serological tests is essential for healthcare professionals, researchers, and policymakers. Given that Coronavirus disease (COVID-19) is treated as an endemic disease in many countries coupled with the widespread availability of the vaccines, gaining an in-depth understanding of SARS-CoV-2 antibody titers in vaccinated population and the performance of the serological assays against COVID-19 becomes exceedingly important. 

Numerous studies have assessed the performance of various SARS-CoV-2 serological assays since the onset of the pandemic. The overall immune responses have been systematically reviewed as the literature on this novel virus continues to expand [6,7,8]. Individual studies have suggested that, in general, the assays have demonstrated reasonable specificities and yet displayed a wide range of sensitivities, varying from 70% to 100% [9,10,11,12,13,14,15,16]. The initial studies evaluated the assay performance on populations with natural infections. Since the introduction of the vaccines, research has shifted to evaluate the impact of vaccines, primarily focusing on the Pfizer BioNTech BNT162b2 vaccine (referred to as Pfizer vaccine), Moderna mRNA-1273 (referred to as Moderna vaccine), Johnson & Johnsons Ad26.COV2.S (referred to as Johnson & Johnson or J&J vaccine), and other vaccines [17,18,19,20,21,22,23]. In rare instances, the serologic assay performances were reported on a population with more than two commonly used vaccines such as a study on a Polish population after four vaccines (BNT162b2, mRNA-1273, ChAdOx1 nCoV-2019, and Ad26.COV2.S) [24]. This study also explored the impact of demographic status such as age, gender, and vaccination timing on the antibody response [24]. It, however, only evaluated one SARS-CoV-2 IgG assay, and no side effects were included in their analysis. 

Various studies have evaluated the impacts of demographic factors and associations between vaccine side effects and antibody responses. While most studies indicated higher neutralizing antibody levels or immunoglobulin levels in females and younger age populations, some did not report any correlations [25,26,27,28,29]. While some did not report any correlations between antibody titers and vaccine side effects [25], many studies indicated some level of correlation [27,29,30]. Again, these studies primarily use one serological assay to evaluate these effects. 

However, to our knowledge, there has not been a comprehensive comparison to evaluate antibody responses using different anti-S assays within the same cohort of individuals who have received all three commonly used vaccines. As part of the Association for Diagnostics & Laboratory Medicine (ADLM) COVID-19 Immunity Study, this study aims to address this gap by providing a thorough examination of the antibody responses among individuals who have been administrated the Pfizer, Moderna, and Johnson & Johnson vaccines. This study also includes a comprehensive survey covering demographic information, vaccine dose and timing, and associated side effects from the same cohort of individuals. 

In this manuscript, we present a detailed comparison of three serology testing methods for Beckman Access SARS-CoV-2 IgG (referred to as Beckman assay), Ortho Clinical Diagnostics VITROS Anti-SARS-CoV-2 IgG (referred to as Ortho assay), Siemens ADVIA Centaur SARS-CoV-2 IgG (referred to as Siemens), and one anti-N antibody assay: Bio-Rad Platelia SARS-CoV-2 Total Ab assay (referred to as BioRad assay). These assays were made available to this study via the ADLM COVID-19 immunity study. These assays were evaluated in a cohort of samples donated from 698 vaccinated individuals in September 2021 who received the three commonly used vaccines—Pfizer, Moderna, and Johnson & Johnson—at various timeframes. Additionally, a total of 305 age- and gender-matched samples from a sample bank obtained in 2015 were used as pre-COVID-19 samples for the study. The associations between the antibody responses and vaccine side effects were also evaluated. 

This study provides insights into the performances of various SARS-CoV-2 anti-S and anti-N assays, the dynamics of antibody responses over time, across various demographic populations, and their associations with side effects. 

## 2. Methods and Materials

### 2.1. Study Design and Population

This study was conducted as part of the Association for Diagnostics & Laboratory Medicine (ADLM), formerly the American Association for Clinical Chemistry (AACC) COVID-19 Immunity Study. Participants were recruited from the members of this laboratory professional association and attendants of its 2021 annual scientific meeting. The study was approved by the University of Maryland Institutional Review Board (submission number: HP-00097761) and informed consent was acquired from all participants. Onsite sample collection happened during the annual scientific meeting between 27 and 30 September 2021. To meet the conference requirements, all participants were fully vaccinated, i.e., with two doses of Pfizer or Moderna or one dose of J&J vaccine. Some also had one dose of booster at the time of sample collection. The samples collected from this study were designated as “COVID-19” exposed samples, which were used to evaluate the assays’ reactive rates or sensitivity. The planning and the sample collection process were recently published [31].

To evaluate the non-reactive rates or specificity of the assays, samples from the AACC 2015 sample bank [32] were utilized. A total of 305 age- and gender-matching samples were retrieved, based on an a priori power calculation. These samples were designated as “pre-COVID-19” samples. 

In addition, this 2021 study incorporated an online survey that took place between 9 September and 20 October 2021 to gather participants’ demographic and general health information, COVID-19 vaccination records, and associated side effects. The summary of the survey was recently published [33].

### 2.2. Specimen Collection and Storage

Information regarding the specimen collection process has recently been made available [34]. In summary, venous samples were collected into a serum separator tube (BD 368774) and sera were collected after clotting and centrifugation. Serum samples were then transported to the Centers for Disease Control and Prevention within four hours for aliquoting (350 μL per aliquot) on TECAN (Mannedorf, Switzerland) and stored at −80 °C until analysis.

### 2.3. Sample Analysis

One set of serum aliquots was sent to the University of Maryland for antibody analysis. The three anti-S assays were Beckman Access SARS-CoV-2 IgG (Beckman Coulter, Inc., Brea, CA, USA), Siemens ADVIA Centaur SARS-CoV-2 IgG (Siemens Healthcare Diagnostics Inc., Tarrytown, NY, USA), and Ortho Clinic Diagnostics VITROS Anti-SARS-CoV-2 IgG (Ortho Clinical Diagnostics Inc., Rochester, NY, USA). Another set of serum aliquots was analyzed at a national reference lab for anti-N antibodies based on the Bio-Rad Platelia SARS-CoV-2 Total Ab assay (Bio-Rad Laboratories, Hercules, CA, USA). These assays were referred to as Beckman, Siemens, Ortho, and BioRad assays (assay details see below). All assays received FDA’s EUA, the samples were analyzed as per manufacturers’ instructions [35,36,37,38], and the assays are summarized below. All the reagents were supplied by the manufacturers specifically for this study. All samples were tested once.

### 2.4. Anti-SARS-CoV-2 Spike (Anti-S) or Receptor Binding Domain (Anti-RBD) Antibody Assays

#### 2.4.1. Beckman

The Beckman Access SARS-CoV-2 IgG assay is a two-step enzyme chemiluminescent paramagnetic particle immunoassay used for the qualitative detection of IgG antibodies to SARS-CoV-2 protein specific to the receptor-binding domain (RBD) of the S1 protein in human serum or plasma (dipotassium EDTA, tripotassium EDTA, lithium heparin, and sodium citrate). Test results are reported with signal to calibrator (S/CO) values as non-reactive (≤0.80 S/CO), equivocal (>0.80 to <1.00 S/CO), or reactive ≥ 1.00 S/CO). Equivocal samples were excluded from sensitivity and specificity calculations.

#### 2.4.2. Siemens

The Siemens ADVIA Centaur^®^ SARS-CoV-2 IgG assay is a two-step sandwich chemiluminescent (indirect) immunoassay used for the qualitative and semi-quantitative detection of IgG antibodies to the SARS-CoV-2 protein specific to the RBD of the S1 protein in human serum or plasma (lithium heparin). Assay results for this test are reported in Index Values and as non-reactive (<1.00 Index) or reactive (≥1.00 Index). 

#### 2.4.3. Ortho

The Ortho VITROS Anti-SARS-CoV-2 IgG assay is a two-stage immunometric assay used for the quantitative detection of IgG antibodies to the SARS-CoV-2 spike protein S1 in human serum or plasma (EDTA and lithium heparin). Numerical results in Binding Antibody Unites (BAU) per mL are displayed with a non-reactive (<17.8 BAU/mL) or reactive (≥17.8 BAU/mL).

### 2.5. Anti-SARS-CoV-2 Nucleocapsid (Anti-N) Antibody Assay

#### Bio-Rad

The Bio-Rad Platelia SARS-CoV-2 total antibody is a one-step antigen (Ag) capture enzyme-linked immunosorbent assay used for the qualitative detection of total anti-SARS-CoV-2 nucleocapsid antibodies (IgM/IgG/IgA) in human serum or plasma (dipotassium EDTA, tripotassium EDTA, lithium heparin, ACD, or sodium citrate). Specimen ratios are calculated using the optical density (OD) of the specimen (Specimen OD) divided by the mean Ods for the cut-off control R4 (OD_M_R4). Results for specimen ratios are reported as negative (<0.8), equivocal (≤0.8 to <1.0), or positive (≥1.0).

### 2.6. Data Analysis

The subjects were defined as “previously infected” if they had self-reported a COVID-19 infection before the survey or if they self-reported a previous positive test for SARS-CoV-2. A total of 83 subjects self-reported a COVID-19 infection and 39 reported a previous positive test for SARS-CoV-2. Another 8 self-reported as not previously having COVID-19 but reported a positive test. Therefore, a total of 91 subjects were classified as “previously infected” in this study.

Three more previously infected subjects had also received a booster dose of vaccine at the study time; these were excluded from analyses that isolated previously infected samples.

For receiver operating characteristic curve (ROC) analysis and calculation of positive predictive value (PPV) and negative predictive value (NPV), true positive samples were vaccinated for COVID-19 while true negative samples were from pre-COVID-19 samples and had negative anti-S assays. PPV was defined as true positives/(true positives + false positives) × 100%, and NPV was defined as true negatives/(true negatives + false negatives) × 100%. Sensitivity was calculated as true positives/(true positives + false negatives). Specificity was calculated as true negatives/(true negatives + false positives). Spearman correlations were measured between each anti-S and anti-N assay and the demographic and clinical variables (Table S3). Linear models of log-anti-S and log-anti-N assay levels were fit for each covariate and clinical variable. These models were fit for all vaccinated subjects and separately for only subjects with Pfizer or Moderna vaccines and no previous infection or booster for the anti-S assays. The anti-N model includes only subjects with a previous infection. The multiplicative coefficients for each model were calculated and plotted in a forest plot.

## 3. Results

### 3.1. Participants Demographics

A total of 698 samples from volunteers alongside 305 selected pre-COVID-19 samples, were analyzed using the four assays. Due to reagent or technical issues, the results from all four assays were obtained for 580 of the 698 COVID-19 samples and 247 of the 305 pre-COVID-19 samples; only these samples were included for analysis in this paper (Table S1). 

The median age for COVID-19 and pre-COVID-19 samples was 50 (IQR = 39–59) and 41 (IQR = 32–53), respectively, with slightly more female than male participants (Table 1). Over 75% of participants in both groups were white and 14% identified as Hispanic. Among the COVID-19 samples, 57.9% completed vaccination with Pfizer, followed by Moderna (30.2%) and J&J (7.4%). A total of 26 (4.3%) participants received vaccines other than the three commonly used ones. Half of the participants had their last dose of vaccine more than 5.5 months (174 days) before the study and 51.6% reported experiencing some level of side effects with 47.4% describing them as moderate and 4.1% as severe (Table 1).

**Figure 1:**
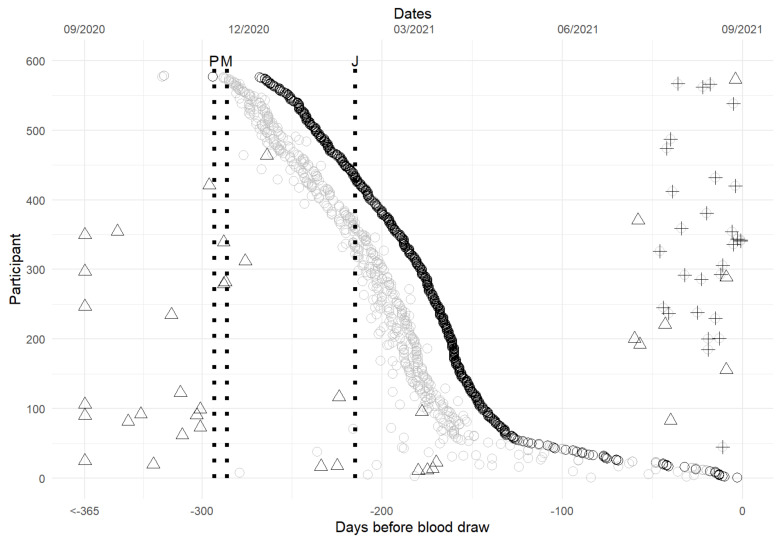
Retrospective dates of first vaccine (grey), second vaccine (black), booster (+), and positive COVID-19 test (triangle). Day 0 is the date of blood collection, and the subjects are sorted by their days since completing vaccination (black outline). Dotted vertical lines indicate the introduction of Pfizer (P), Moderna (M), and Johnson & Johnson (J) vaccines. The top X-axis indicates the calendar dates and the lower X-axis indicates the relative days to the date of blood collection.

Two participants took the AstraZeneca and J&J vaccines in November 2020, while the rest were vaccinated after the EUA of the Pfizer (11 December 2020), Moderna (18 December 2020), or J&J (27 February 2021) vaccines (Figure 1). The time since the last dose of vaccination ranged from 1 to 322 days with most samples taken 5–7 months post-vaccination. Participants who received a two-dose vaccine typically received their second dose four weeks after the first dose, and 26 participants received booster doses at the time of the study. Participants who completed Pfizer and Moderna vaccinations had similar distributions of time since vaccination, with the majority having completed it 150 days before the study. Those who completed J&J vaccination mostly had their vaccination earlier, approximately 180 days before the study (Figure S1). Thirty-eight (38) participants reported the date of a positive diagnostic test with the majority of them (n = 30) reporting it before their vaccination, while a few (n = 8) reported it within two months of the study (Figure 1).

### 3.2. Assay Performance for Study Population

#### 3.2.1. Anti-S Assays

Among the 580 COVID-19 samples, Beckman, Ortho, and Siemens reported 575, 566, and 562 reactive (positive) results, translating to sensitivities of 99.5%, 97.6%, and 96.9%, respectively (Table 2A). The agreements between these assays were 98.1% (Ortho vs. Beckman), 99.3% (Ortho vs. Siemens), and 97.4% (Beckman vs. Siemens) for the COVID-19 samples (Table S2). All three assays reported 562 (96.9% of COVID-19) reactive results and 18 samples had one or more non-reactive results. Three samples tested negative for all three anti-S assays, and clinical history from the survey did not provide clear reasons for these three negatives. Two more were equivocal for Beckman and negative for the other assays. Nine were negative for Ortho and Siemens and four tested negative by only Siemens.

For the 247 pre-COVID-19 samples, Beckman, Ortho, and Siemens anti-S assays showed 215, 247, and 246 non-reactive results, translating to specificities of 90%, 100%, and 99.6%, respectively (Table 2A). The agreements between these assays for these samples were 87.0% (Ortho vs. Beckman), 99.6% (Ortho vs. Siemens), and 86.6% (Beckman vs. Siemens). 

We evaluated if medical conditions contributed to the false positives of the testing. All 247 described themselves as “generally healthy”, of which 9 (3.6%) had recent muscle/skeletal injury, 83 (33.6%) were on medication, 39 (15.8%) had high cholesterol, 74 (30.0%) had an immediate family member(s) with known heart disease, and 38 (15.4%) were current or former smokers. Of the false positives (32 Beckman + 1 Siemens), 0 had recent muscle/skeletal injury, 10 (30.3%) were on medication, 6 (18.2%) had high cholesterol, 7 (21.2%) had an immediate family member(s) with known heart disease, and 3 (9.1%) were current or former smokers. Furthermore, we evaluated if there were potential cross relativities to the SARS-CoV-1 virus that occurred in Asia that could be a cause of the false positives. Among the 247 samples, 27 (11%) were Asian/Pacific Islander/Indian and 11 (4%) resided in Asia (China, South Korea, Indonesia, Japan, Singapore, Australia, and New Zealand). Beckman, with the most positive results from this group, had 32 positive or equivocal results, of which 4 (12.5%) were Asian and 2 (6%) resided in Asia. No compelling evidence suggests that either a medical condition or the geographic location of the pre-COVID-19 samples is linked to the false positive results.

The anti-S results from these three assays exhibited varying levels of performance for different vaccines. Participants who received the Moderna (n = 150) vaccine demonstrated 100% sensitivity from all three assays. Participants who received the Pfizer (n = 267) vaccine showed more than 99% sensitivity from all assays. And there were no significant differences in the sensitivity among the three assays for participants who received Pfizer or Moderna vaccines. However, participants who received the J&J (n = 27) vaccine indicated lower sensitivities ranging from 70.4% (Siemens) to 81.5% (Ortho) and 96.3% (Beckman), and the sensitivity from Beckman was significantly higher than Siemens (*p* = 0.01) (Table 2B). This lower sensitivity for J&J could be attributed to its overall low antibody levels compared to Moderna and Pfizer vaccines (see Impact of Vaccine Types on Antibody Levels). All three assays indicated 100% sensitivity for participants who had booster (n = 26) and 93.4% to 96.7% sensitivity for participants who self-reported having a previous infection (n = 91). 

#### 3.2.2. Anti-N Assay

The Bio-Rad anti-N assay was the only anti-N assay available to be evaluated for this study and was assessed for the 91 participants who self-reported previous infections. This assay indicated 46.2% sensitivity and 99.2% specificity based on the 91 COVID-19 participants and 247 pre-COVID-19 participants. Of the 47 subjects who reported a previous positive test for SARS-CoV-2, a higher sensitivity of 59.6% (28 BioRad positive) was observed. The assay was not sensitive enough to indicate previous COVID-19 infection. Nonetheless, the assay demonstrated good specificity to rule out SARS-CoV-2 infection with only one equivocal and one positive result out of 247 pre-COVID-19 samples.

**Figure 2:**
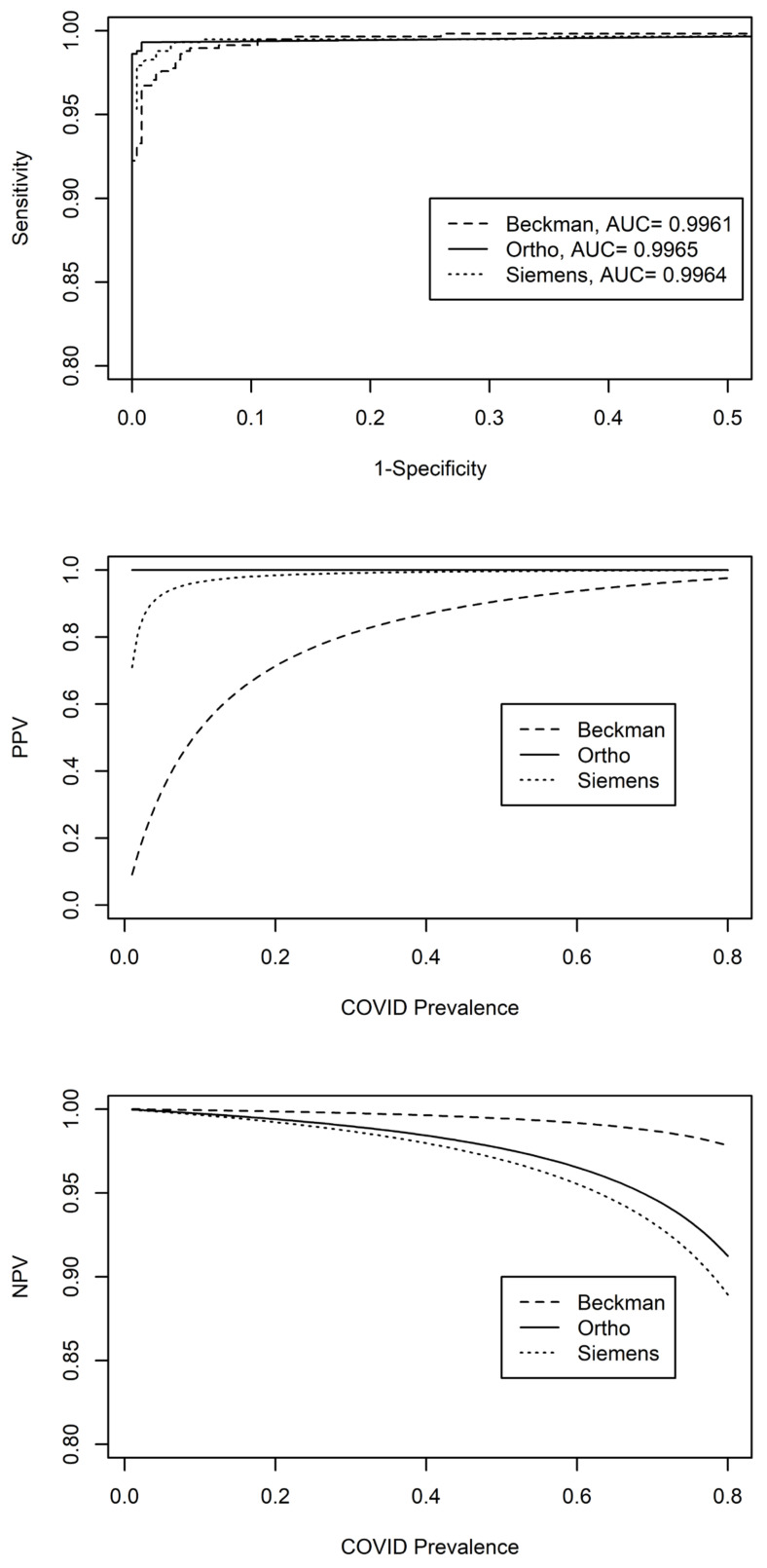
ROC prediction curves and the range of positive and negative predictive values over COVID-19 prevalence for each assay.

The predictive value of an assay based on sensitivity and specificity is intricately tied to the prevalence of the disease, which has undergone significant changes throughout the pandemic. The negative predictive values (NPVs) were all more than 99% until the prevalence reached 33%, at which point Siemens dropped to 98% NPV (Figure 2). However, the positive predictive values (PPVs) differed significantly among these three assays with Beckman being the lowest, particularly when the prevalence is less than 15% (Figure 2).

At the prevalence of 1%, Beckman, Siemens, and Ortho had 9.1%, 71%, and 100% PPV, respectively. These values quickly increased to 52.5%, 96.4%, and 100% PPV when the prevalence reached 10% (Figure 2). The PPVs increased with a higher prevalence of COVID-19 and surpassing 90% for the Siemens assay at 5% of prevalence while the Beckman assay did not exceed 90% PPV until nearly a 50% prevalence of COVID-19 (Figure 2). The low PPVs from Beckman were directly linked to its low specificity at 90% compared to 100% and 99.6% for Ortho and Siemens assays, respectively. Sensitivities at 90% can result in significant disadvantages in assessing PPV at a lower disease prevalence stage. When the sensitivity and specificity were considered together, Ortho had the highest area under the curve followed by Siemens and then Beckman (Figure 2).

#### 3.2.3. Impact of Vaccine Types on Antibody Levels

Participants received only Pfizer (n = 267), Moderna (n = 150), and J&J (n = 27) vaccines as well as participants with booster doses (n = 26) and previous infections (n = 91) were used to assess the impact of the vaccine types on quantitative assay levels.

In general, the five groups exhibited significant differences for all four assays (*p* < 0.0001). The three vaccines (Pfizer, Moderna, J&J) showed significant differences for the anti-S (*p* < 0.0001) but not for the anti-N (*p* = 0.102) assay. 

**Figure 3:**
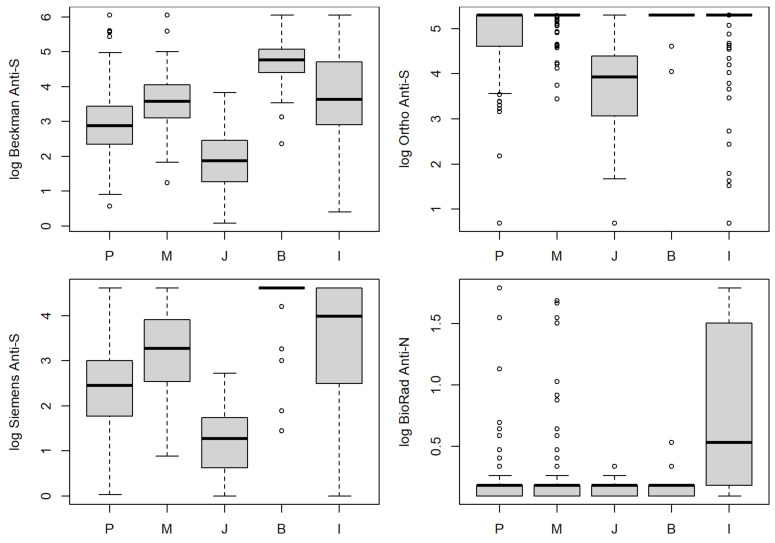
Antibody levels measured by anti-S and anti-N assays in the blood for different vaccination types. P—Pfizer, M—Moderna, J—Johnson & Johnson, B—vaccinated and Booster dose, and I—previously Infected.

The median anti-S levels from patients who had the Moderna vaccine were highest (39, 200 BAU/mL, 28 for Beckman, Ortho, Siemens, respectively), followed by those who received Pfizer (20, 200 BAU/mL, 13 for Beckman, Ortho, and Siemens) and J&J vaccines (8, 65 BAU/mL, 3 for Beckman, Ortho, and Siemens) for all three anti-S assays. Many values exceeded the upper end of the reporting range for the Ortho assay after Pfizer or Moderna vaccines (Figure 3). Booster doses and natural infection significantly increased the overall antibody levels based on the Beckman and Siemens assays (Figure 3). Regardless of the vaccine type, very few participants had positive results from the anti-N antibody. However, participants who self-reported previous infection had significantly higher anti-N antibody levels than those who did not (*p* < 0.0001, Figure 3).

### 3.3. Impact of Demographics and Side Effects on Antibody Levels

**Figure 4:**
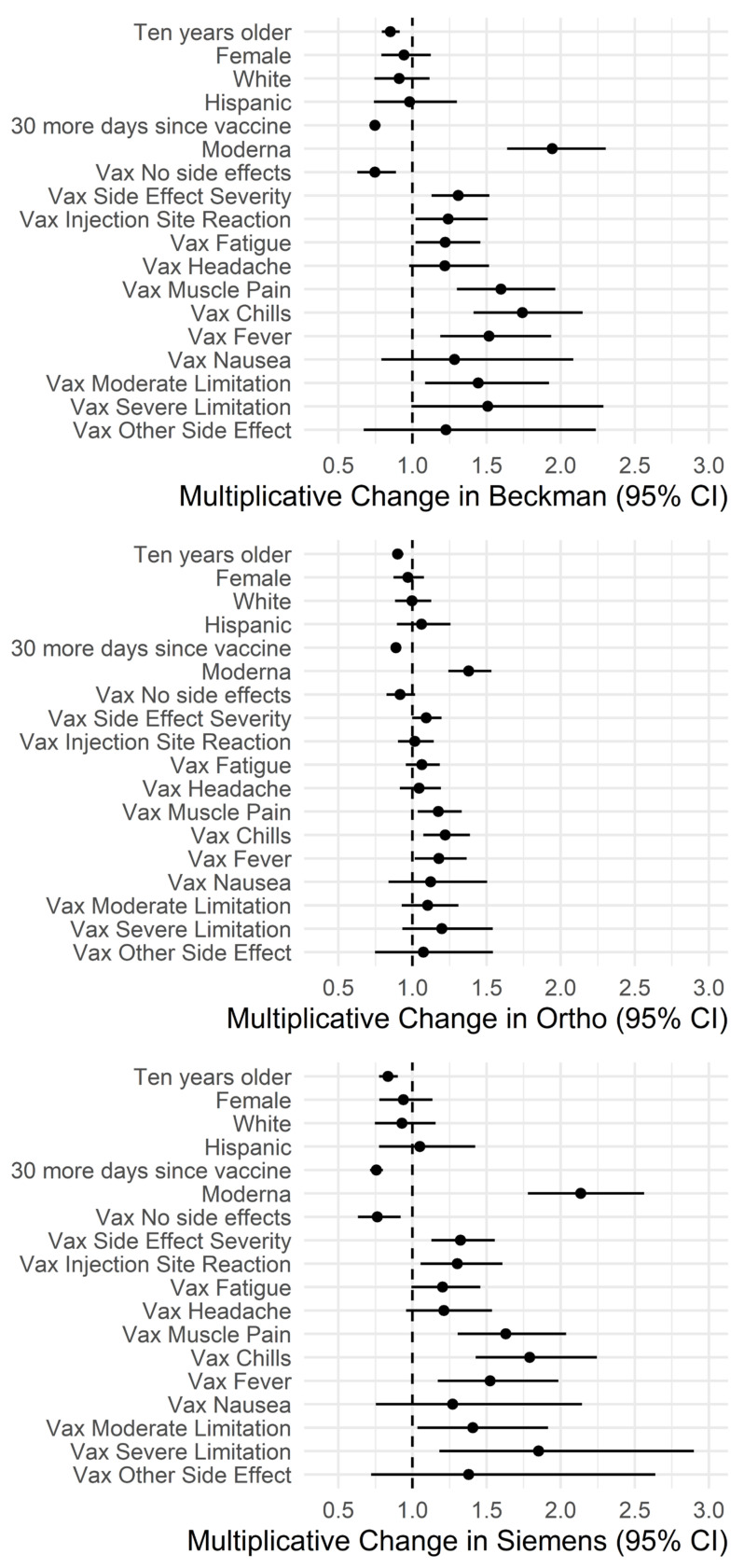
Effects of demographic characteristics and vaccine side effects on antibody levels for the anti-S. The changes are multiplicative and models use only subjects with Pfizer or Moderna vaccine and no previous infection or booster for the anti-S assays. The BioRad model includes only subjects with a previous infection.

Multivariate analysis indicated that anti-S levels from three assays showed similar patterns for age, sex, race, ethnicity, and days since the last vaccine dose. Younger age and closer to the last vaccine dose were associated with higher antibody levels in the participants (Figure 4, Table S3). No significant differences were observed between antibody levels among male and female participants when various vaccines were considered separately (Figure S2), and the same held true for race and ethnicity groups. None of the demographic information evaluated showed significant correlations with anti-N antibody levels.

The three anti-S assays indicated the same direction of associations with side effects, but not the same significance levels. All three assays demonstrated that higher levels of antibody levels were significantly associated (*p* < 0.05) with experiencing any side effects and more severe side effects, as well as more muscle pain, chills, fever, and moderate limitations (*p* < 0.05, Figure 4). Anti-N antibody levels only indicated a significant correlation with headache (Table S3) which disappeared when only considering participants who had previous infection (Figure 4, Table S3). 

### 3.4. Impact of Age and Time since Vaccine on Antibody Levels

**Figure 5:**
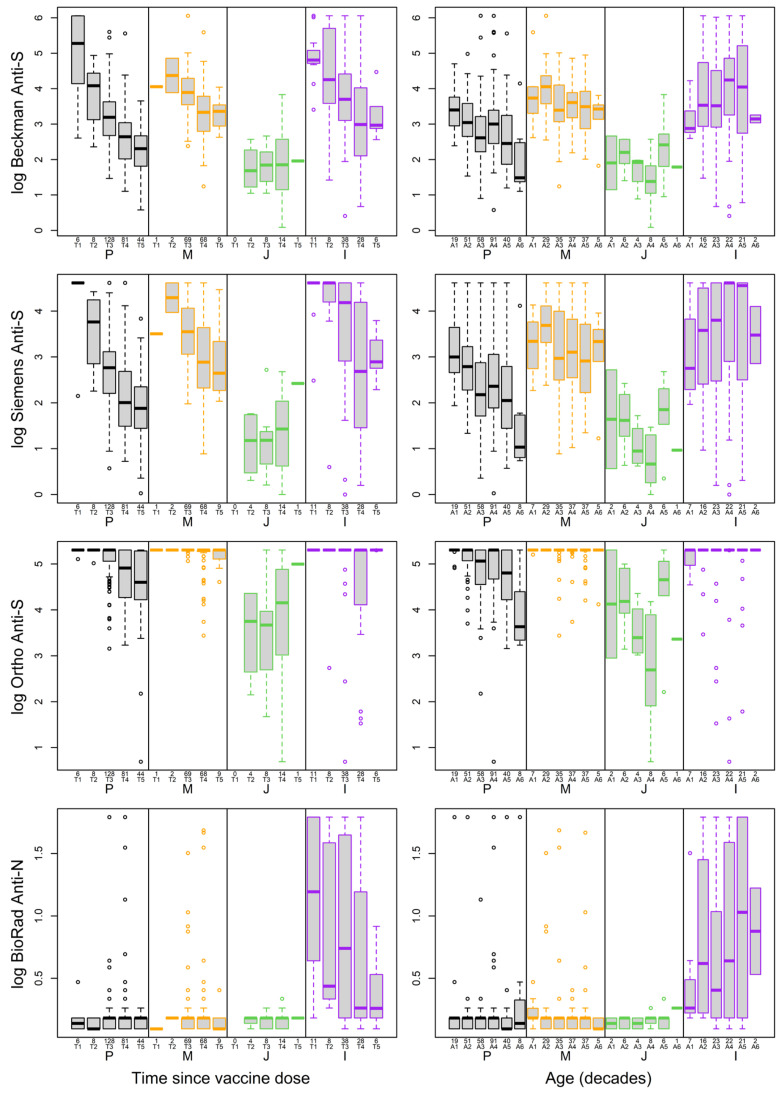
Impact of time since last vaccine (**left**) and age (**right**) for vaccine types on antibody levels assessed by the three anti-S and one anti-N assay. The time since the last vaccine dose was delineated as T1: <2 months; T2: 2–4 months; T3: 4–6 months; T4: 6–8 months; T5: >8 months. The age is grouped as A1: ≥ 20 < 30; A2: ≥ 30 < 40; A3: ≥ 40 < 50; A4: ≥ 50 < 60; A5: ≥ 60 <70 ; A6: ≥ 70 < 85. Vaccine types are referred to as P—Pfizer, M—Moderna, J—Johnson & Johnson, and I—previously Infected.

Anti-S antibody levels exhibited a negative correlation with the days since the last vaccine dose across all three anti-S assays (Figure 4, Table S3). Antibody levels displayed similar patterns over time relative to the last dose of vaccine for the three vaccines, with Beckman and Siemens being the most similar to each other (Figure 5, left side). Anti-S antibody levels peaked soon after the Pfizer vaccination and then exhibited a declining trend over time. Participants vaccinated within four months showed high anti-S levels that slowly declined over four to ten months. Anti-S antibodies after the J&J vaccine remained stable between 2 and 10 months during the study period. Participants with natural infection and vaccination showed a decrease in anti-S antibodies over time since vaccination.

Anti-S antibody levels were negatively correlated with age across all three anti-S assays (Figure 4, Table S3). The results from all three assays indicated that the amount of anti-S antibodies dropped significantly with age for Pfizer (*p* < 0.0005, Figure 5, right side). Antibody levels dropped significantly with age for those with the Moderna vaccine (*p* < 0.05) based on Beckman and Siemens assay results, but not for the Ortho assay (*p* = 0.30). The amount of anti-S antibodies did not significantly change with age for participants who took the J&J vaccine or those who were previously infected (0.22 < *p* < 0.85). The amount of anti-N was not significantly different based on the time since infection for any group (0.51 < *p* < 0.88). However, overall anti-S and anti-N levels in participants who had the J&J vaccine were lower than those who had other vaccines and were higher in participants who were previously infected than in vaccinated participants (Figure 5).

## 4. Discussion

The goals of the study were to assess the performances of three SARS-CoV-2 anti-S and one anti-N serological assay, exploring the associations of the antibody levels with demographic information, timing of vaccination, and vaccine side effects in one cohort of 580 COVID-19 samples and 247 pre-COVID-19 samples. Our studies indicated varying performances among the serological assays with sensitivities ranging from 96.9% to 99.5% and specificities ranging from 90% to 100%, which resulted in significant variations for PPVs across disease prevalence levels, underscoring the impact of prevalence on the interpretation of positive test results for each assay. No patterns were identified among the samples that were negative for one or more of the anti-S assays, and conditions such as cardiovascular disease, transplants, diabetes, overweight, cancer, gastrointestinal conditions, respiratory tract conditions, lung disease, thalassemia, neurological disease, and autoimmune were indicated from the survey. Similarly, reviewing the positive results from the pre-COVID-19 samples did not reveal clear indications for the sample being positive. 

Interestingly, the same assay showed different sensitivity and specificity for different vaccines with the most sensitivity associated with the Moderna vaccine, followed by the Pfizer vaccine. The variations in performances were correlated with overall antibody levels with Moderna being the highest, followed by Pfizer and J&J. These performance variations resulted in significant differences for predictive values particularly PPVs throughout the pandemic when the disease had various prevalences, suggesting consideration of serological assays and vaccine types when choosing an assay for immune response monitoring after vaccination.

The variability of serological assays aligned with the existing literature [39], and our findings of higher antibody levels after the Moderna vaccine were consistent with previous reports [40]. Despite assay variability, all exhibited reasonable sensitivity, particularly when considering individuals after Pfizer and Moderna vaccines for both Beckman and Siemens assays. This indicated the effectiveness of these two assays in detecting the antibody levels after vaccination. It was not surprising to find that antibody levels were the lowest after the J&J vaccine as reported in previous studies comparing mRNA vaccine versus viral vector-based vaccine [41]. These low values resulted in reduced apparent sensitivity for the serological assays. This study did not intend to evaluate the efficacy of these vaccines, but we have reported lower neutralizing antibody levels in participants who received the J&J vaccine [34], and it has been reported that a single dose of the J&J vaccine had lower efficacy than Pfizer and Moderna vaccines [42]. 

The Ortho assay displayed a unique pattern, with a significant number of participants reaching the high end of the reportable range, making it challenging to detect a correlation with age or timing of vaccinations. This contrasts with the other two anti-S assays and warrants further investigation, as it may impact the assay’s utility. Nonetheless, the high sensitivity from the Ortho assay indicated by our study is consistent with previous findings [43,44]. This underscores the importance of evaluating assay performances before conducting studies and making informed choices regarding the selection of assays for specific research objectives. It is important to highlight that the Ortho assay is marketed as a semi-quantitative assay, while both Beckman and Siemens assays are qualitative. In this study, the antibody levels were assessed based on the instrument’s quantitative results.

The study’s retrospective nature limited the assessment of antibody levels to a two-month time frame revealing that antibody levels peaked within two months after the Pfizer and J&J vaccines and within two to four months after the Moderna vaccine. The antibody levels did not peak within the first two months as previously reported for Moderna [45,46], which might be due to the fact that only one sample was obtained within two months of the last dose of vaccine for our cohort. The declining trends for Pfizer and Moderna and the relatively stable trend for the J&J vaccine were consistent with previous reports [41,45,46]. 

A major strength of this study was assessing the serological assay performances and the impacts of demographic information and side effects on antibody levels in the same cohort. The findings suggested that higher antibody levels were associated with younger age, closer proximity to the last vaccine dose, and individuals experiencing any side effects and more severe side effects, along with more muscle pain, chills, fever, and moderate limitations. In contrast to a previous study indicating the association between sex and more severe side effects [33], sex was not the deciding factor for the antibody levels, suggesting that the association between female and more severe side effects was influenced by factors beyond stronger immune response. One study based on the population of 225 Japanese participants indicated the rise of neutralizing antibody levels on day 28 after the first dose of the Pfizer vaccine and moderate reverse correlations were found between the neutralizing antibody levels and age while no correlation was found with side effects [25]. Similar studies were performed between neutralizing antibodies and the Pfizer vaccine in a Mexican population [26]. A study from Israel indicated that higher antibody levels were associated with severe side effects and no gender differences were found after the Pfizer vaccine [29]. A study in a cohort of Italian healthcare professionals demonstrated significantly higher antibody titers after the Pfizer vaccine in females and younger age groups and they also reported more frequent side effects in females than in males [27]. SARS-CoV-2 immunoglobulin G and total antibody levels, neutralizing activity, and avidity exhibited negative correlations with age in patients aged 1 to 24 years [28]. In general, the literature suggested that younger age was associated with higher antibody levels and more and/or severe side effects. 

The study has several limitations including its survey nature, with only one time point for sample collection, and retrospectively obtaining survey information, potentially resulting in recall bias. Efforts were made to address the inconsistency of reported vaccine times. In addition, only one anti-N assay was assessed due to limitations in partnership with manufacturers partnerships and we could not perform comparisons to other anti-N assays in the market at the time. The study cohort, requiring full vaccination for the 2021 AACC (now ADLM) annual scientific meeting, limited the evaluation of anti-N antibody performances. In addition, many of the samples had previous infections reported more than 240 days (8 months) before the sample collection. Various reported values of the half-life of anti-N ranged from 68 days [47], 71 days [48] to 85 days [49] and significant variation was observed in the reported studies among individuals. This may result in less sensitivity of anti-N antibodies to the population in this study. However, anti-S half-lives were estimated to be more than 180 days which led to a measurable amount of 1.5–2 years after infection [50,51,52]. In this study, among the 38 positive results reported with test dates, there were 22 subjects whose positive reports were at least 240 days before the time of the blood draw and 30 subjects whose positive reports were at least 150 days before the blood draw (Figure 1). Of these, 5 out of 22 (23%) and 7 out of 30 (23%) had negative anti-N results. We did not see a clear correlation between the anti-N negative rate and the infection time before the sample collection. However, our sample size was small for previously infected samples with known dates to draw a definite conclusion. Despite these limitations, the study provides valuable insights into the complexities of serological assay performance and antibody dynamics post-vaccination.

## 5. Conclusions

All anti-S serological assays demonstrated adequate performance, although larger variations were evident among them, particularly in their specificities and reportable ranges. The specificity ranged from 90% to 100% which resulted in significant variations for positive and negative predictive values. Care should be taken when applying these assays in clinical and epidemiological studies and interpreting them under various CVID prevalence conditions. Antibody levels were shown to be vaccine-dependent, resulting in various assay performances for different vaccines. The overall antibody levels reached their highest soon after the vaccination and demonstrated a declining trend over time for Moderna and Pfizer vaccines while the J&J vaccine displayed a relatively low overall antibody level that was stable up to our study time frame of 10 months. These discrepancies warrant caution for the medical and scientific community when choosing serological assays for various purposes. Factors to consider include the purpose of the study, the stage of the pandemic, and the disease prevalence. 

Our findings underscored that younger age, proximity to the last vaccine dose, and the experience of any side effects, particularly severe ones, were associated with higher antibody levels. These insights are crucial for healthcare providers and researchers, offering valuable guidance in selecting the most appropriate serology testing method tailored to specific needs. We believe that the knowledge presented in this manuscript serves as a valuable reference for ongoing efforts in the development and refinement of serological testing methods, not only for COVID-19 but also for emerging infectious diseases in the future.

## Figures and Tables

**Table 1:** Demographic information of the study population.

		COVID-19 Samples	Pre-COVID-19 Samples
N		580	247
Age ^a^		50 (39–59)	41 (32–53)
Gender (Male %)		270 (46.6%)	111 (44.9%)
Race (White %)		437 (75.3%)	187 (75.7%)
Ethnicity (Hispanic/Latino %)		81 (14.0%)	31 (12.6%)
Vaccine type	Pfizer	336 (57.9%)	-
	Moderna	175 (30.2%)	-
	J&J	43 (7.4%)	-
	Others ^b^	26 (4.5%)	-
Days since last dose of vaccine ^a^		174 (148–214)	-
Any side effects (%)		299 (51.6%)	-
Moderate limitations (%)		275 (47.4%)	-
Severe limitations (%)		24 (4.1%)	-

^a^: median (IQR), the rest are shown as n (%). ^b^: other vaccines include 1 Unvaccinated, 1 Unknown, 1 Sputnik, 1 Covaxin, 5 Sinopharm, 13 AstraZeneca, 2 Pfizer (only 1 dose), 1 Moderna (only 1 dose), and 1 J&J followed by Moderna.

**Table 2:** Predictive performance of the anti-S assays for the study population and different vaccine types.

		COVID-19 ^a^	Pre-COVID-19	Sensitivity	Specificity
**A. Overall Population**					
Beckman ^b^	Pos	575	24	99.5%	90.0%
	Neg	3	215		
Ortho	Pos	566	0	97.6%	100%
	Neg	14	247		
Siemens	Pos	562	1	96.9%	99.6%
	Neg	18	246		
**B. Breakdown by Vaccine Types ^c^**					
Beckman					
Pfizer (n = 267)	Pos	266	24	99.6%	90.0%
Moderna (n = 150)	Pos	150		100%	
J&J (n = 27)	Pos	26		96.3%	
Booster (n = 26)	Pos	26		100%	
Prev. Infection (n = 91)	Pos	88		96.7%	
Ortho					
Pfizer (n = 267)	Pos	265	0	99.3%	100%
Moderna (n = 150)	Pos	150		100%	
J&J (n = 27)	Pos	22		81.5%	
Booster (n = 26)	Pos	26		100%	
Prev. Infection (n = 91)	Pos	85		93.4%	
Siemens					
Pfizer (n = 267)	Pos	264	1	98.9%	99.6%
Moderna (n = 150)	Pos	150		100%	
J&J (n = 27)	Pos	19		70.4%	
Booster (n = 26)	Pos	26		100%	
Prev. Infection (n = 91)	Pos	85		93.4%	

^a^: COVID-19 total n = 580, Pre-COVID-19 total n = 247; ^b^: cut-offs for non-reactive results: Beckman ≤ 0.8 (n = 10 equivocal are excluded); Ortho < 17.8; Siemens < 1; ^c^: pre-COVID-19 samples did not have breakdowns for vaccines, and the overall positive results and specificity are identical to the overall population values.

## Data Availability

Data is available upon request.

## Supplementary Materials

The following supporting information can be downloaded at: https://www.mdpi.com/article/10.3390/v16020292/s1, Figure S1: Distribution of Participants Over Days Since Second (Pfizer and Moderna) or Last Dose (J&J) of Vaccination; Figure S2: Anti-S and Anti-N Results with Subgroups for Vaccine Types Over Sex (M/F). The differences between males and females are not significant. Table S1: Summary of Samples Tested by Assays; Table S2: Agreement of Antibody Levels (reactive vs non-reactive) Between the Three Anti S Assays; Table S3: Correlations Between Antibody Levels and Participants’ Demographic information and Vaccine Side Effects.

## Abbreviations

Association for Diagnostics & Laboratory Medicine (ADLM), American Association for Clinical Chemistry (AACC), Emergency Use Authorization (EUA), severe acute respiratory syndrome coronavirus 2 (SARS-CoV-2), and coronavirus disease-19 (COVID-19).
