# Supplementary material for: Performance of Three Anti-SARS-CoV-2 Anti-S and One Anti-N Immunoassays for the Monitoring of Immune Status and Vaccine Response
Source: Viruses. 2024 Feb 14;16(2):292. doi: 10.3390/v16020292 (PMC10891747; doi:10.3390/v16020292)

Supplemental Materials

Table S1. Summary of Samples Tested by Assays

Antibody	Manufacturer	Description	COVID Samples	Pre-COVID Samples
Anti-S	Beckman	Access SARS-CoV-2 IgG	589	305
Anti-S	Siemens	ADVIA Centaur SARS-CoV-2 IgG (COV2G)	597	247
Anti-S	Ortho	VITROS Anti-SARS-CoV-2 IgG Quantitative	592	305
Anti-N	Bio-Rad	Platelia SARS-CoV-2 Total Ab assay	695	305
		Number of Samples Having Results from All Assays	580	247

Table S2. Agreement of Antibody Levels (reactive vs non-reactive) Between the Three Anti S Assays

	Siemens	Ortho
COVID Samples		
Beckman	97.4%	98.1%
Siemens		99.3%
Pre-COVID samples		
Beckman	86.6%	87.0%
Siemens		99.6%
All Samples		
Beckman	94.2%	94.8%
Siemens		99.4%

Table S3. Correlations Between Antibody Levels and Participants' Demographic information and Vaccine Side Effects [&]

Assay Types/ Demographic info and Side Effects		Anti-S			Anti-N	Anti-N
		Beckman	Siemens	Ortho	All	PrevInf
Age		-0.11	**-0.11**	**-0.15**	-0.03	0.12
Sex		-0.02	-0.01	-0.02	0.03	0.05
Race		0.00	0.01	0.03	-0.05	-0.15
Ethnicity		0.04	0.02	0.01	-0.07	-0.09
Days since last dose		**-0.49**	**-0.45**	**-0.36**	**-0.11**	**-0.38**
No side effects		**-0.15**	**-0.12**	-0.10	-0.03	-0.03
Side effect severity		**0.15**	**0.13**	0.11	0.03	0.02
Individual side effects (Y/N)	Injection site	**0.14**	**0.13**	0.06	0.01	0.08
	Fatigue	0.10	0.08	0.06	-0.01	-0.02
	Headache	0.08	0.09	0.06	**0.11**	0.01
	Muscle pain	**0.17**	**0.13**	0.11	0.06	0.08
	Chills	**0.19**	**0.17**	**0.15**	0.03	-0.02
	Fever	**0.15**	**0.15**	0.11	0.06	0.04
	Nausea	0.01	0.00	0.00	-0.05	-0.01
	Moderate limitation	**0.13**	**0.12**	0.09	0.03	0.05
	Severe limitation	0.06	0.08	0.07	0.02	-0.11
	Other	0.08	0.08	0.05	0.04	0.14

[&] Only included participants with American vaccines or previous infections
Bold: statistically significant with $p < 0.01$

Figure S1: Distribution of Participants Over Days Since Second (Pfizer and Moderna) or Last Dose (J&J) of Vaccination.

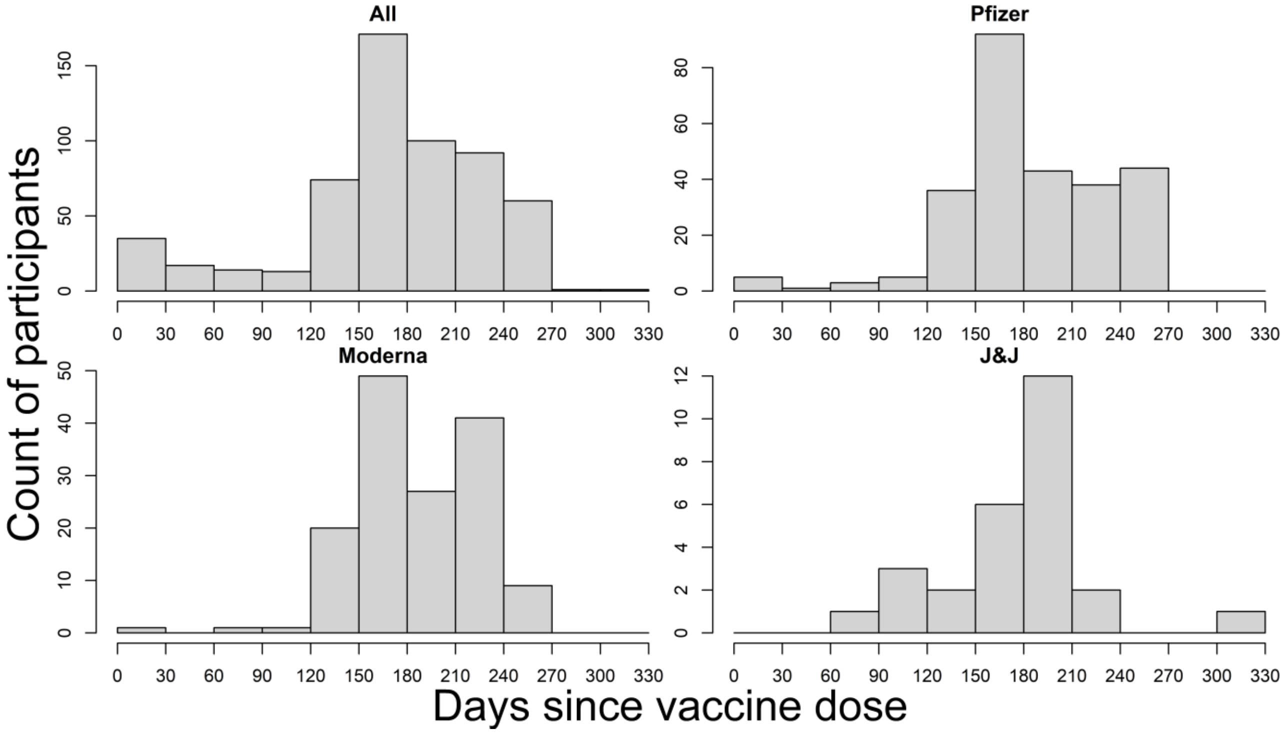

Figure S2: Anti-S and Anti-N Results with Subgroups for Vaccine Types Over Sex (M/F). The differences between males and females are not significant.

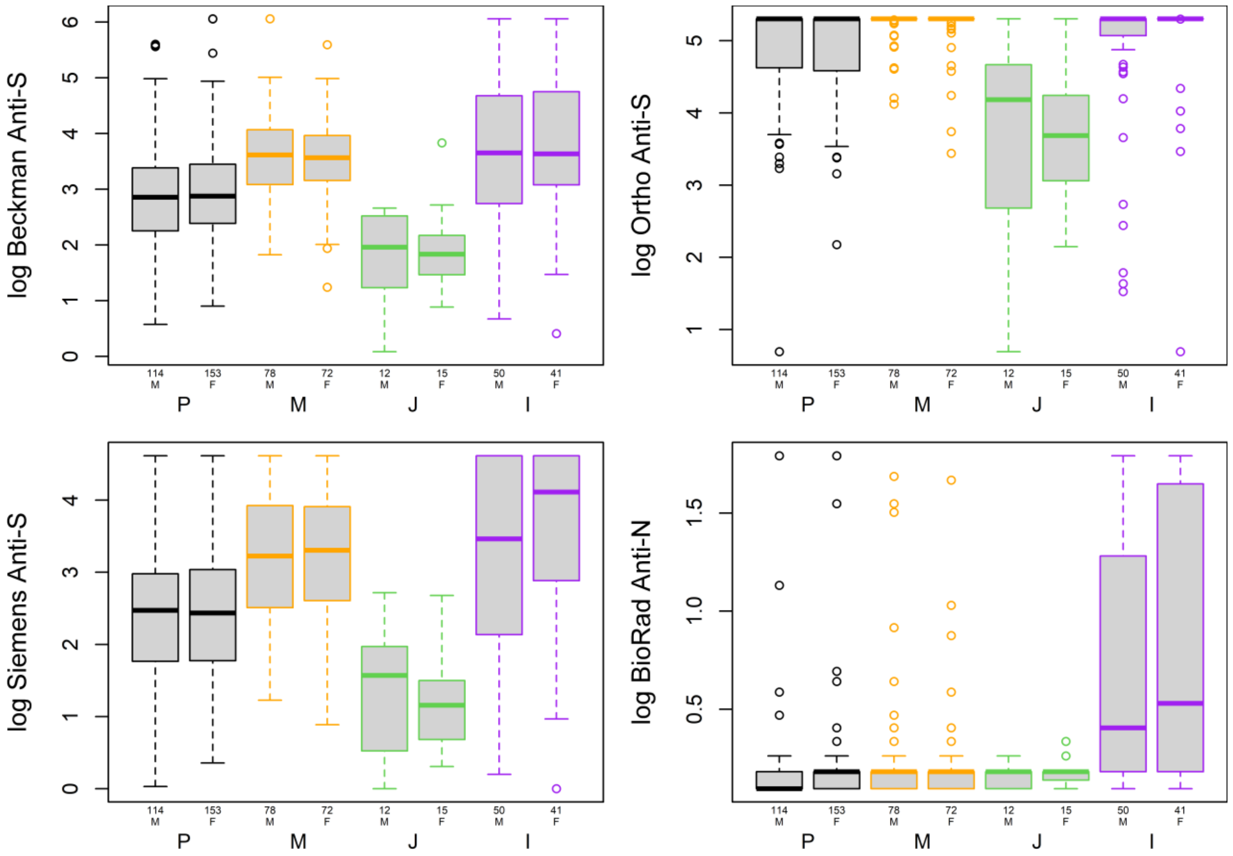

Supplement: Supplementary file 1 [file viruses-16-00292-s001.zip › viruses-2855676-supplementary.pdf]
